# Supplementary figures and images for: Synergistic apoptotic effects in cancer cells by the combination of CLK and Bcl-2 family inhibitors
Source: PLoS One. 2020 Oct 16;15(10):e0240718. doi: 10.1371/journal.pone.0240718 (PMC7567398; doi:10.1371/journal.pone.0240718)

S1 Fig

A

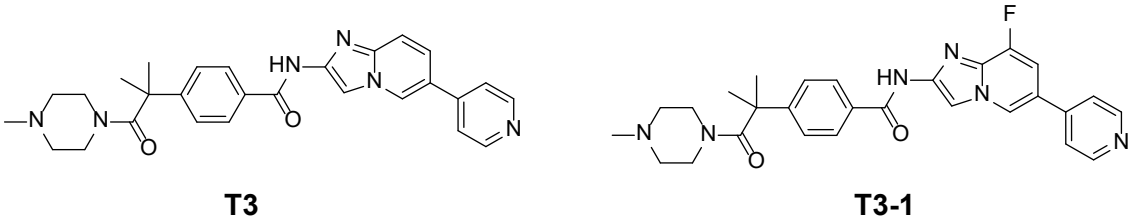

B

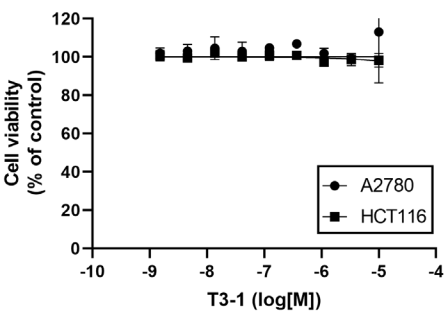

Supplement: S1 Fig — (A) Chemical structures of T3 and T3-1. (B) Viabilities of A2780 and HCT116 cells normalized to the DMSO control after 72 h of T3 treatment. The x-axis shows compound concentration (logM). The y-axis shows percent inhibition of adenosine triphosphate content, compared to the DMSO control. Data are presented as mean ± standard deviation (SD) of three independent experiments. (PDF) [file pone.0240718.s001.pdf]
