# Supplementary material for: Synergistic apoptotic effects in cancer cells by the combination of CLK and Bcl-2 family inhibitors
Source: PLoS One. 2020 Oct 16;15(10):e0240718. doi: 10.1371/journal.pone.0240718 (PMC7567398; doi:10.1371/journal.pone.0240718)
Supplement: S1 File — (PDF) [file pone.0240718.s002.pdf]

S2 File. Overview of the synthesis of T3-1 (*N*-(8-fluoro-6-(pyridin-4-yl)imidazo[1,2-a]pyridin-2-yl)-4-(2-methyl-1-(4-methylpiperazin-1-yl)-1-oxopropan-2-yl)benzamide trihydrochloride)

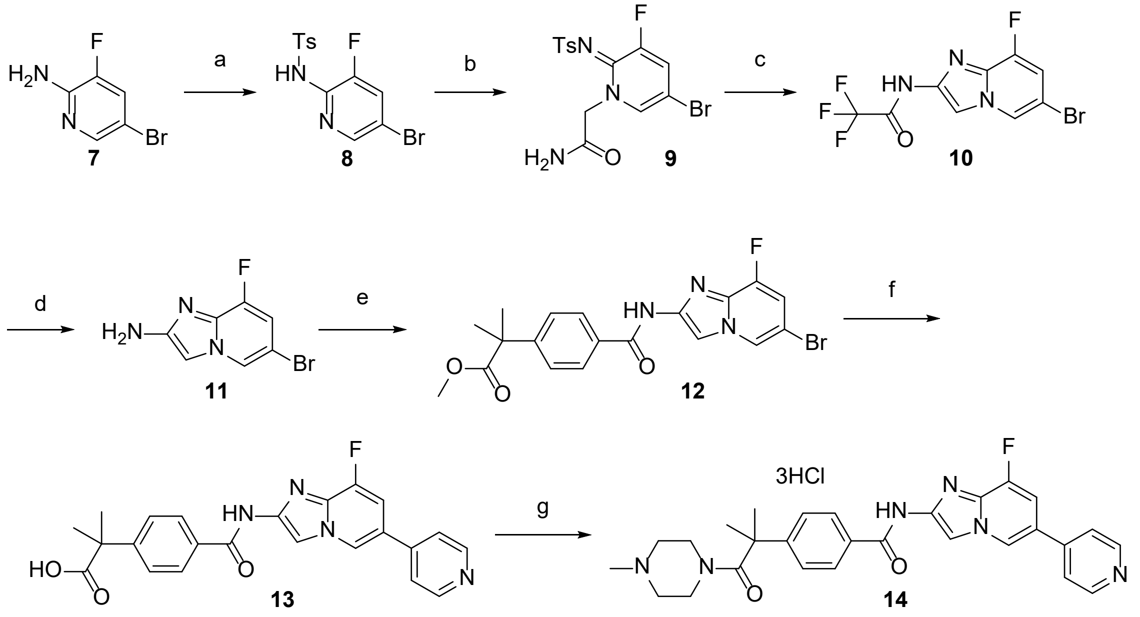

Reagents and conditions: a. i) *p*-toluenesulfonyl chloride, pyridine; ii) NaOH; b. iodoacetamide, *N*,*N*-diisopropylethylamine; c. trifluoroacetic anhydride; d. HCl; e. i) 4-(1-methoxy-2-methyl-1-oxopropan-2-yl)benzoic acid, oxalyl chloride, *N*,*N*-dimethylformamide; f. i) 4-(4,4,5,5-tetramethyl-1,3,2-dioxaborolan-2-yl)pyridine, [1,1'-bis(diphenylphosphino)ferrocene]palladium (II) dichloride dichloromethane adduct, cesium carbonate; ii) NaOH; g. 1-methylpiperazine, 1-[Bis(dimethylamino)methylene]-1*H*-1,2,3-triazolo[4,5-b]pyridinium 3-oxide hexafluorophosphate, *N*,*N*-diisopropylethylamine.

Synthesis of T3-1 (14)

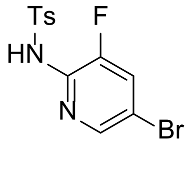

8: *N-(5-bromo-3-fluoropyridin-2-yl)-4-methyl-N-tosylbenzenesulfonamide*

To a solution of 5-bromo-3-fluoropyridin-2-amine (compound **7** in Fig. S2, 8.8 g, 46.07 mmol) in anhydrous pyridine (100 ml), *p*-toluenesulfonyl chloride was slowly added (17.56 g, 92.15 mmol) at 0°C. The reaction mixture was then heated at 90°C for 16 h. Pyridine was removed under reduced pressure to afford the crude product. To a solution of this product in a solution of methanol (300 ml) and water (50 ml) was added NaOH (7.4 g in 25 ml of water). The reaction mixture was stirred at room temperature for 3 h. Methanol was removed under reduced pressure.

The residue was diluted with water and adjusted to pH~5-6 with 1N HCl. The mixture was then extracted with AcOEt. The organic layer was washed with water, brine, dried over sodium sulfate, and concentrated to give the title compound (15.0 g, 94%).

^{1}H-NMR (DMSO-d_6): δ: 2.28 (3H, s), 7.13 (2H, d, J = 8.0 Hz), 7.35 (1H, dd, J = 10.4, 2.0 Hz), 7.62-7.67 (3H, m).

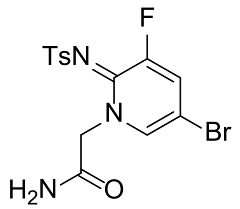

9: *2-(5-bromo-3-fluoro-2-(tosylimino)pyridin-1(2H)-yl)acetamide*

To a solution of **8** (13.8 g, 40.0 mmol) in dry *N*,*N*-dimethylformamide (150 ml) was added *N*,*N*-diisopropylethylamine (15.48 g, 120.0 mmol) and iodoacetamide (14.8 g, 40.0 mmol). The mixture was stirred at room temperature for one day. This reaction mixture was diluted with water, and extracted with AcOEt. The organic layer was washed with sat. ammonium chloride solution, brine, dried over magnesium sulfate and concentrated to obtain the crude product that was then chromatographed on SiO_2 with AcOEt to afford the title compound (11.94 g, 74%).

^{1}H-NMR (DMSO-d_6) δ: 2.42 (3H, s), 4.15 (2H, s), 7.06 (1H, s), 7.40-7.44 (3H, m), 7.55 (2H, d, J = 8.4 Hz), 8.34-8.36 (2H, m).

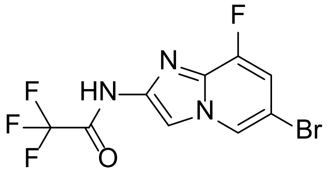

10: *N-(6-bromo-8-fluoroimidazo[1,2-a]pyridin-2-yl)-2,2,2-trifluoroacetamide*

A suspension of **9** (11.94 g, 29.7 mmol) in a mixture of dichloromethane (150 ml) and trifluoroacetic anhydride (80 ml) was heated at reflux for 3 h. The solvent was removed under reduced pressure and the residue partitioned between AcOEt and sat. $NaHCO_3$ solution. The organic layer was washed with 1N HCl, water and brine, and dried over Na_2SO_4. The solvent was removed under reduced pressure and the residue was washed with ether to afford the title compound (5.85 g, 60%).

^{1}H-NMR (DMSO-d_6) δ: 7.54 (1H, d, J = 10.4 Hz), 7.73 (1H, s), 8.62(1H, s), 11.75 (1H, brs).

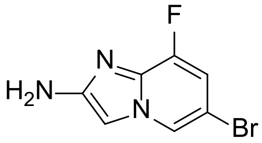

11: *6-bromo-8-fluoroimidazo[1,2-a]pyridin-2-amine*

To a suspension of **10** (2 g, 6.13 mmol) in methanol (45 ml) was added 2N HCl (15.34 ml, 30.67 mmol). The mixture was stirred at 50°C for 2 h. The mixture was neutralized with 2N NaOH (14 ml) to ca. pH 7. The mixture was then concentrated, and the residue partitioned between AcOEt and aqueous $NaHCO_3$. The aqueous layer was extracted with AcOEt. Combined organic layers were washed with brine, and concentrated. The residue was triturated with a mixed solution of AcOEt, ether and hexane to give the title compound (1.20 g, 85%).

^{1}H-NMR (DMSO-d_6) δ: 5.42 (2H, s), 6.87 (1H, s), 7.11 (1H, dd, J = 11.0, 1.5 Hz), 8.23 (1H, d, J = 1.5 Hz).

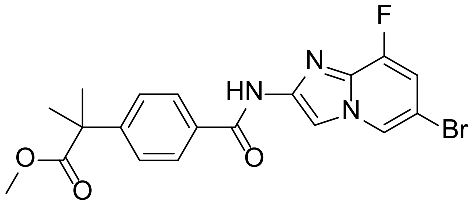

12: *methyl 2-(4-((6-bromo-8-fluoroimidazo[1,2-a]pyridin-2-yl)carbamoyl)phenyl)-2-methylpropanoate*

To a solution of 4-(1-methoxy-2-methyl-1-oxopropan-2-yl)benzoic acid (319 mg, 1.43 mmol) in dry tetrahydrofuran (5 ml) was added oxalyl chloride (0.137 ml, 1.56 mmol) and catalytic amounts of *N*,*N*-dimethylformamide. The mixture was stirred at room temperature for 1 h followed by azeotropic concentration with toluene (5 ml) to give the corresponding acid chloride. To a solution of the acid chloride in *N*,*N*-dimethylacetamide (5 ml) was added **11** (300 mg, 1.30 mmol). The mixture was stirred at room temperature for 3 h. The mixture was partitioned between AcOEt and water. The aqueous layer was extracted with AcOEt. The combined organic layers were washed with brine and concentrated. The residue was chromatographed on NH-SiO_2 with AcOEt/hexane and triturated with ether to give the title compound (544 mg, 96%).

^{1}H-NMR (DMSO-d_6) δ: 1.56 (6H, s), 3.62 (3H, s), 7.46-7.54 (3H, m), 7.68 (1H, s), 8.03 (2H, d, J = 8.4 Hz), 8.50 (1H, d, J = 1.4 Hz), 10.65 (1H, s).

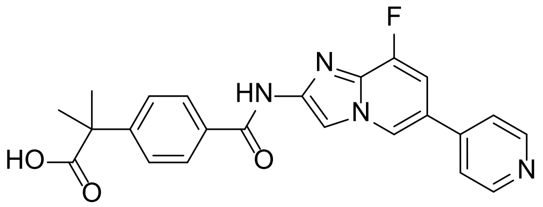

13: *2-(4-((8-fluoro-6-(pyridin-4-yl)imidazo[1,2-a]pyridin-2-yl)carbamoyl)phenyl)-2-methylpropanoic acid*

A mixture of **12** (565 mg, 1.30 mmol), 4-(4,4,5,5-tetramethyl-1,3,2-dioxaborolan-2-yl)pyridine (400 mg, 1.95 mmol), cesium carbonate (635 mg, 1.95 mmol) and [1,1'-bis(diphenylphosphino)ferrocene]palladium(II) dichloride dichloromethane adduct (106 mg, 0.13 mmol) in 1,2-dimethoxyethane (5 ml) and water (0.5 ml) was stirred at 100°C under microwave irradiation for 1 h. The mixture was partitioned between AcOEt and water. The aqueous layer was extracted with AcOEt. The combined organic layers were washed with brine, and passed through a NH-SiO_2 pad (2 g, eluent: 10%MeOH/EtOAc). The filtrate was concentrated, and the residue was triturated with ether to give crude methyl 2-(4-((8-fluoro-6-(pyridin-4-yl)imidazo[1,2-a]pyridin-2-yl)carbamoyl)phenyl)-2-methylpropanoate. To a solution of the crude product in methanol (5 ml) and tetrahydrofuran (2.5 ml) was added 2N NaOH (1.95 ml, 3.90 mmol). The mixture was stirred at 50°C for 8 h. The mixture was neutralized with 2N HCl and extracted with AcOEt. The combined organic layers were washed with brine, dried over Na_2SO_4, and concentrated. The solidified residue was triturated with ether to give the title compound (402 mg, 74 %).

^{1}H-NMR (DMSO-d_6) δ: 1.54 (6H, s), 7.55 (2H, d, J = 8.5 Hz), 7.71-7.79 (2H, m), 7.80-7.86 (2H, m), 8.06 (2H, d, J = 8.3 Hz), 8.61-8.68 (3H, m), 10.70 (1H, s), 12.48 (1H, brs).

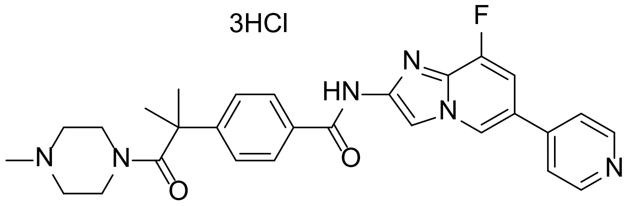

14 (T3-1): *N-(8-fluoro-6-(pyridin-4-yl)imidazo[1,2-a]pyridin-2-yl)-4-(2-methyl-1-(4-methylpiperazin-1-yl)-1-oxopropan-2-yl)benzamide trihydrochloride*

To a mixture of **13** (120 mg, 0.29 mmol), 1-[bis(dimethylamino)methylene]-1*H*-1,2,3-triazolo[4,5-b]pyridinium 3-oxide hexafluorophosphate (164 mg, 0.43 mmol), *N*,*N*-diisopropylethylamine (0.100 ml, 0.57 mmol) and *N*,*N*-dimethylacetamide (4 ml) was added 1-methylpiperazine (0.048 ml, 0.43 mmol). The mixture was stirred at room temperature for 2 h. The mixture was partitioned between AcOEt and brine. The aqueous layer was extracted with

AcOEt. The combined organic layers were washed with brine, and concentrated. The residue was chromatographed on SiO_2 with methanol/AcOEt to give the compound in free form. This product was dissolved in ethanol (2.5 ml), and to the resulting solution was added 4N HCl in AcOEt (0.5 ml) followed by addition of ether (3 ml) to give the title compound (99 mg, 57 %).

^{1}H-NMR (DMSO-d_6) δ: 1.51 (6H, s), 2.69 (3H, d, J = 3.7 Hz), 2.71-3.39 (8H, m), 7.43 (2H, d, J = 8.4 Hz), 7.81 (1H, s), 7.96 (1H, dd, J = 12.5, 1.2 Hz), 8.17 (2H, d, J = 8.4 Hz), 8.38 (2H, d, J = 6.3 Hz), 8.93 (2H, d, J = 6.7 Hz), 9.04 (1H, d, J = 1.1 Hz), 10.98 (1H, s), 11.03 (1H, brs).
