# Supplementary material for: Synergistic apoptotic effects in cancer cells by the combination of CLK and Bcl-2 family inhibitors
Source: PLoS One. 2020 Oct 16;15(10):e0240718. doi: 10.1371/journal.pone.0240718 (PMC7567398; doi:10.1371/journal.pone.0240718)

Supporting information, S3 File

Fig. 2 and Fig. 5A

Western-blot of A2780 and HCT116 cells treated with T3.

The membrane was cut horizontally and western blotted with each antibody as shown in Figure.

Samples order in the frame are shown below.

	A2780						HCT116					
T3 (μM)	0	0	1	1	3	3	0	0	1	1	3	3

Membrane #1:

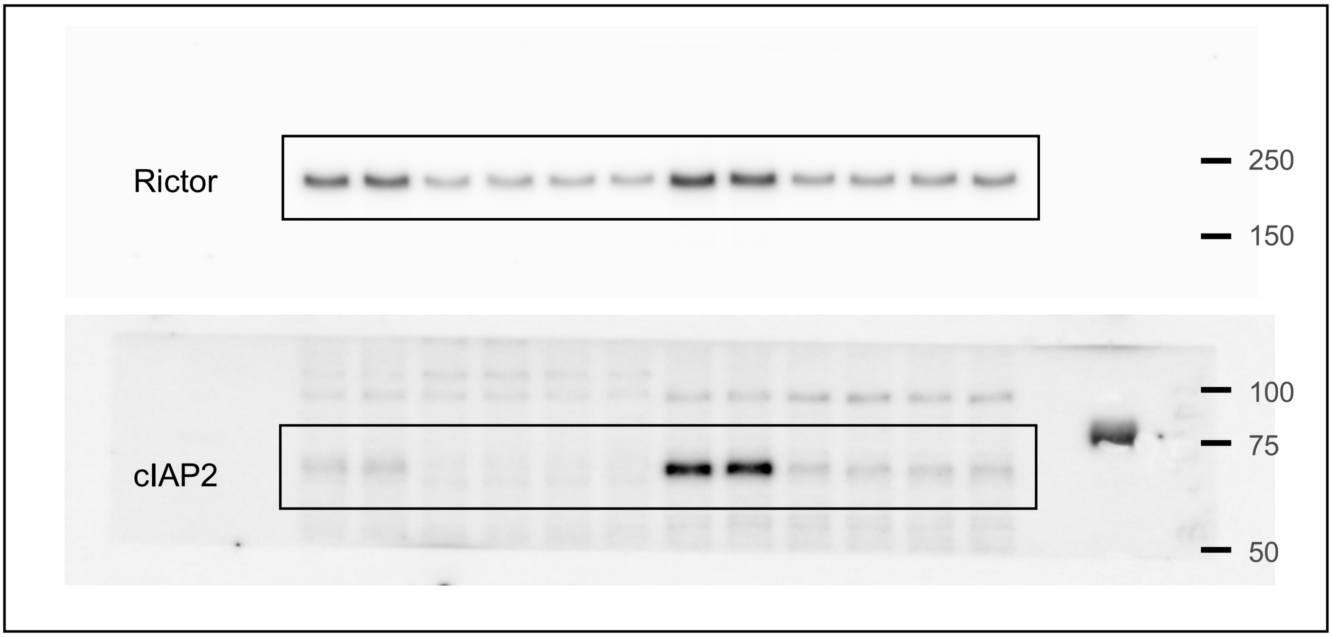

Membrane #2:

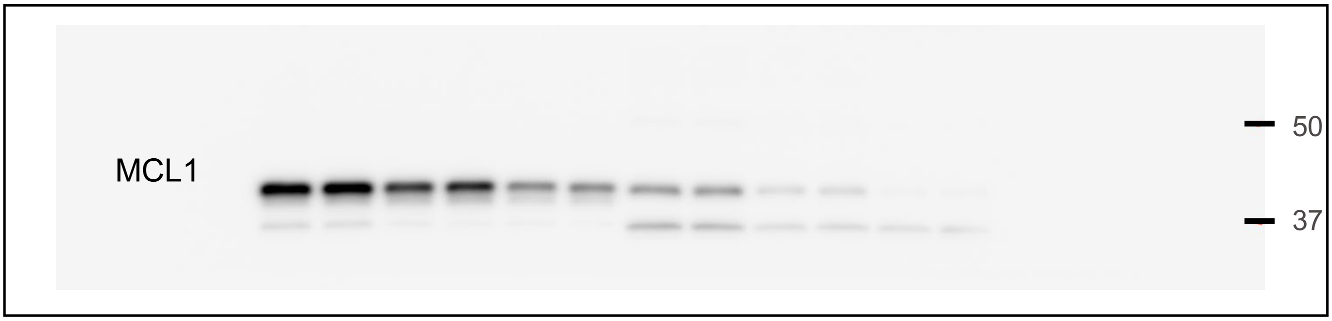

Membrane #3:

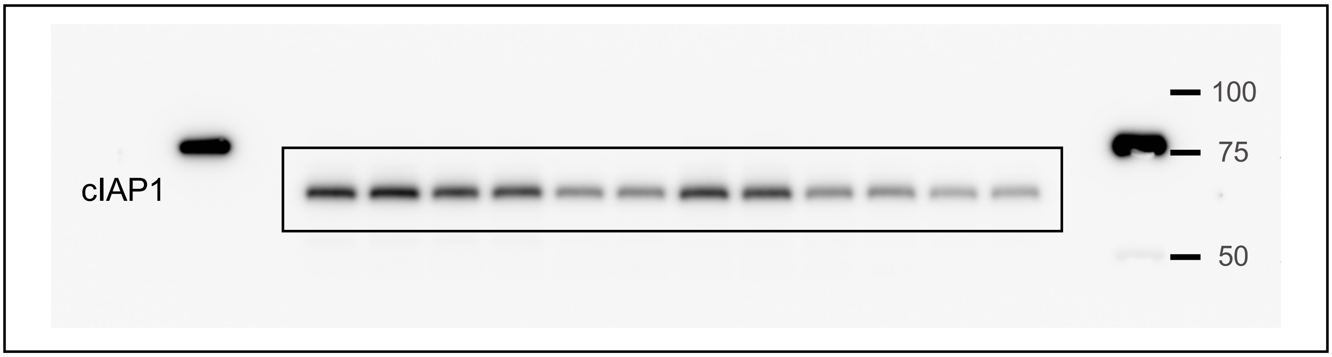

Supporting information, S3 File

Fig. 2 and Fig. 5A (continued)

Western-blot of A2780 and HCT116 cells treated with T3.

The membrane was cut horizontally and western blotted with each antibody as shown in Figure.

Samples order in the frame are shown below.

	A2780						HCT116					
T3 (μM)	0	0	1	1	3	3	0	0	1	1	3	3

Membrane #4:

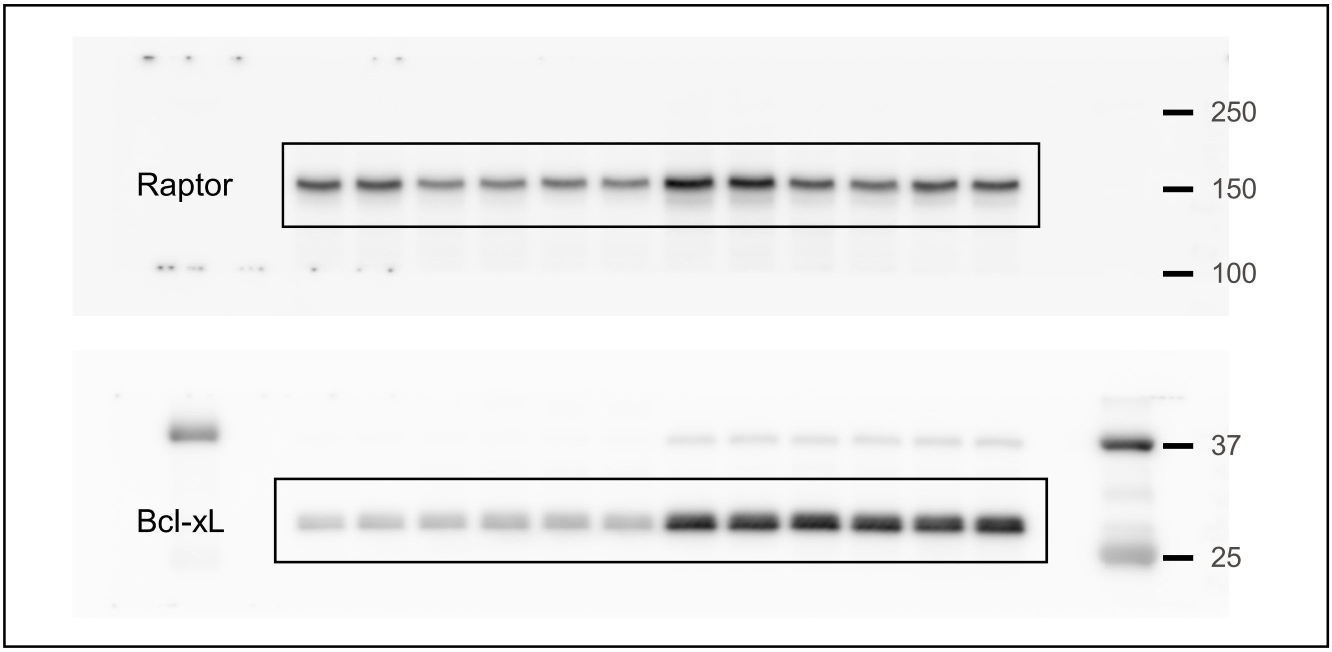

Membrane #5:

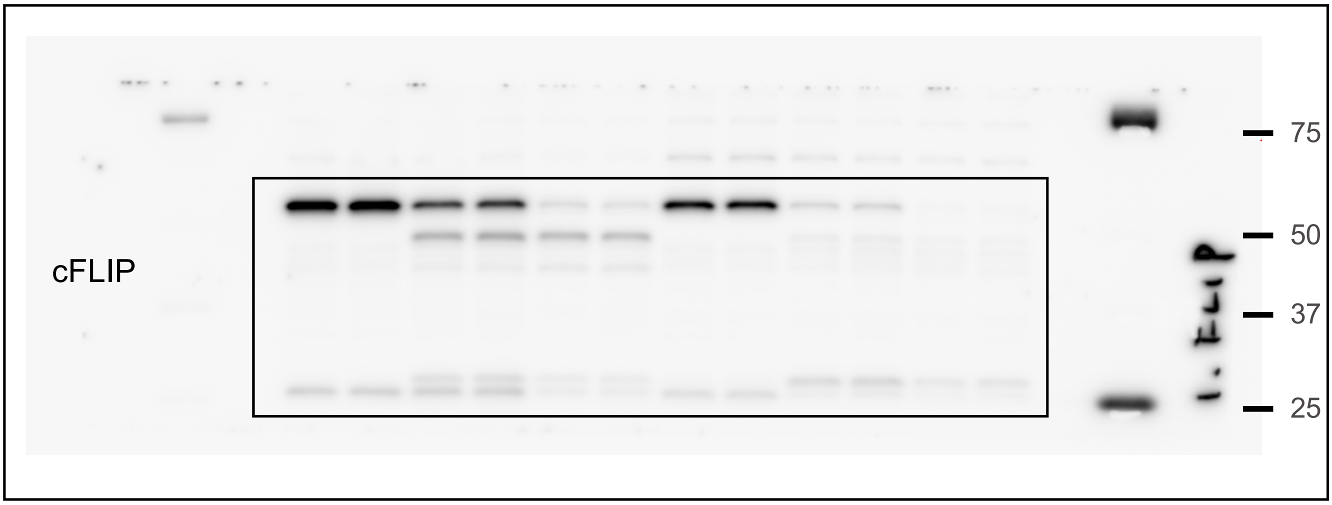

Supporting information, S3 File

Fig. 2 and Fig. 5A (continued)

Western-blot of A2780 and HCT116 cells treated with T3.

The membrane was cut horizontally and western blotted with each antibody as shown in Figure.

Samples order in the frame are shown below.

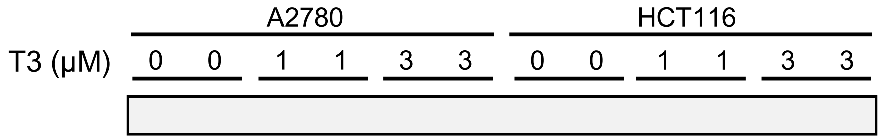

Membrane #6:

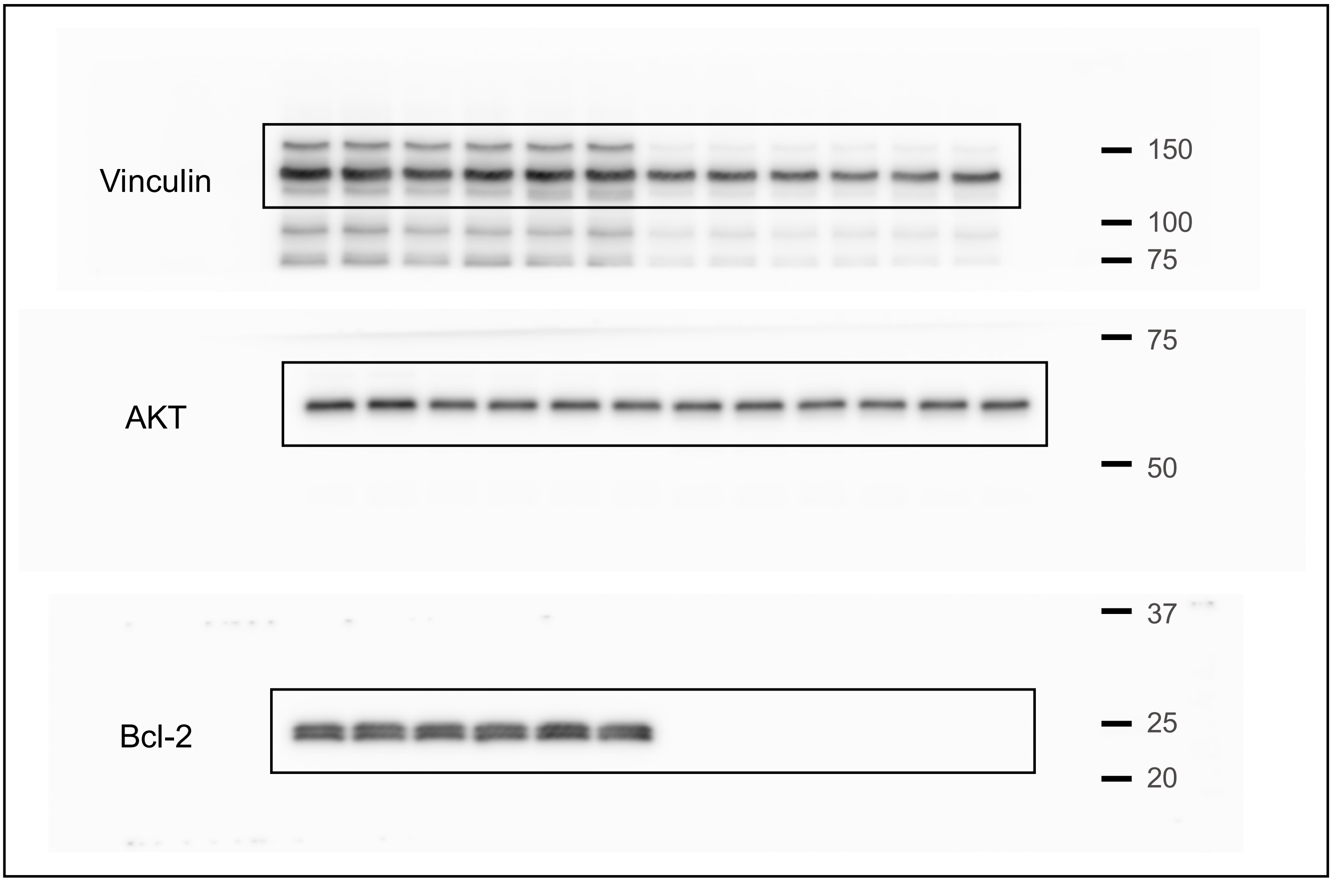

Membrane #7:

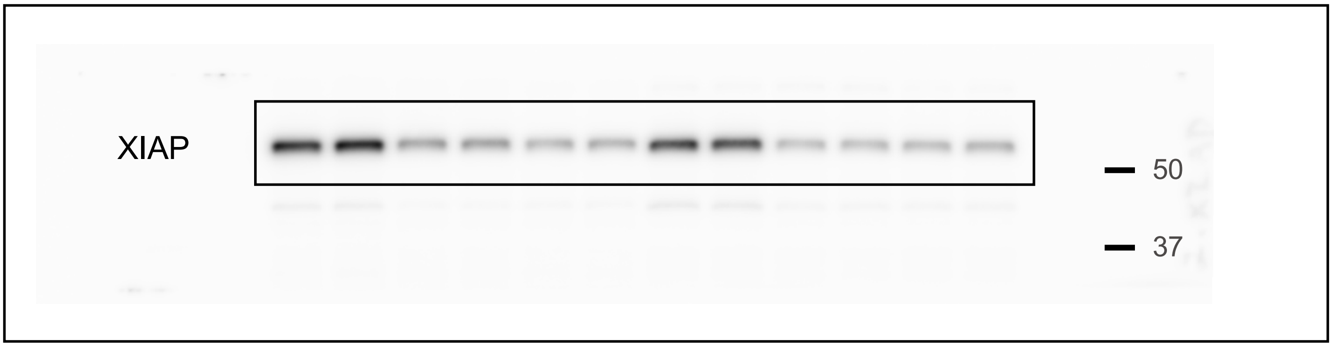

Supporting information, S3 File

Fig. 5B

Western-blot of HCT116 cells treated with T3 and MG132.

The membrane was cut horizontally and western blotted with each antibody as shown in Figure.

Samples order in the frame are shown below.

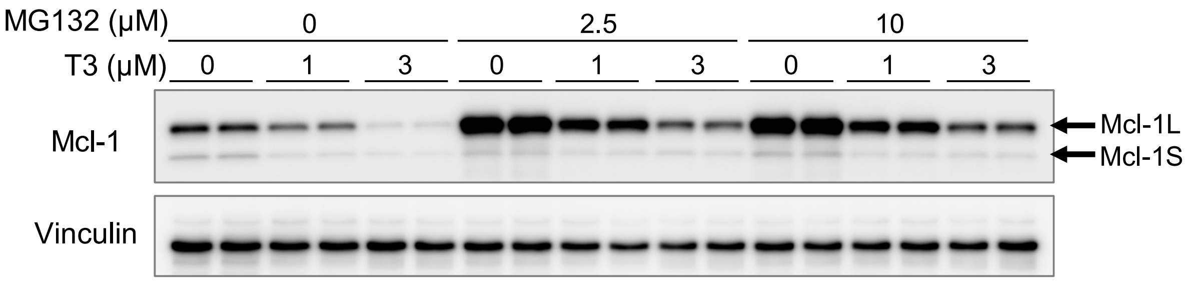

Membrane#8:

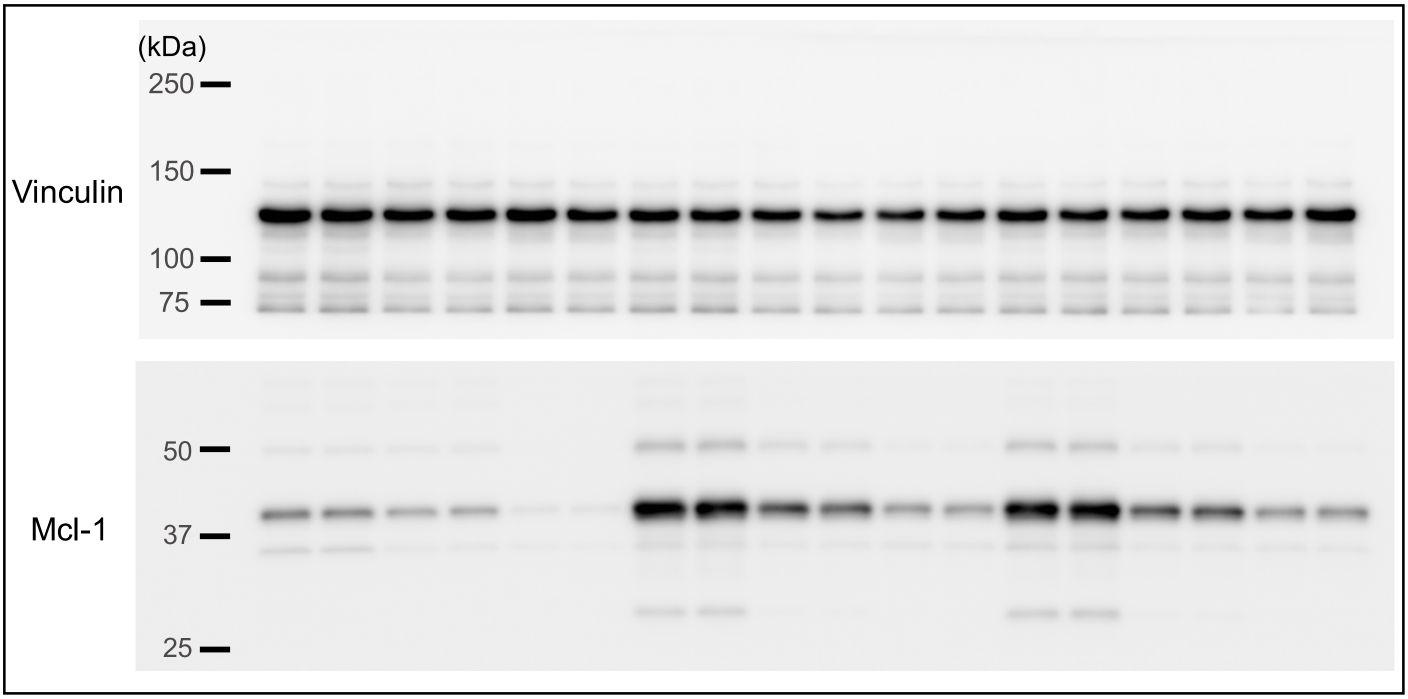

Supplement: S2 File — (PDF) [file pone.0240718.s003.pdf]
