# Supplementary material for: Synergistic apoptotic effects in cancer cells by the combination of CLK and Bcl-2 family inhibitors
Source: PLoS One. 2020 Oct 16;15(10):e0240718. doi: 10.1371/journal.pone.0240718 (PMC7567398; doi:10.1371/journal.pone.0240718)

Supporting information, S4 File

Fig. 4

Raw electropherogram images using capillary electrophoresis LabChip in A2780 and HCT116 cells treated with T3.

CFLAR-L

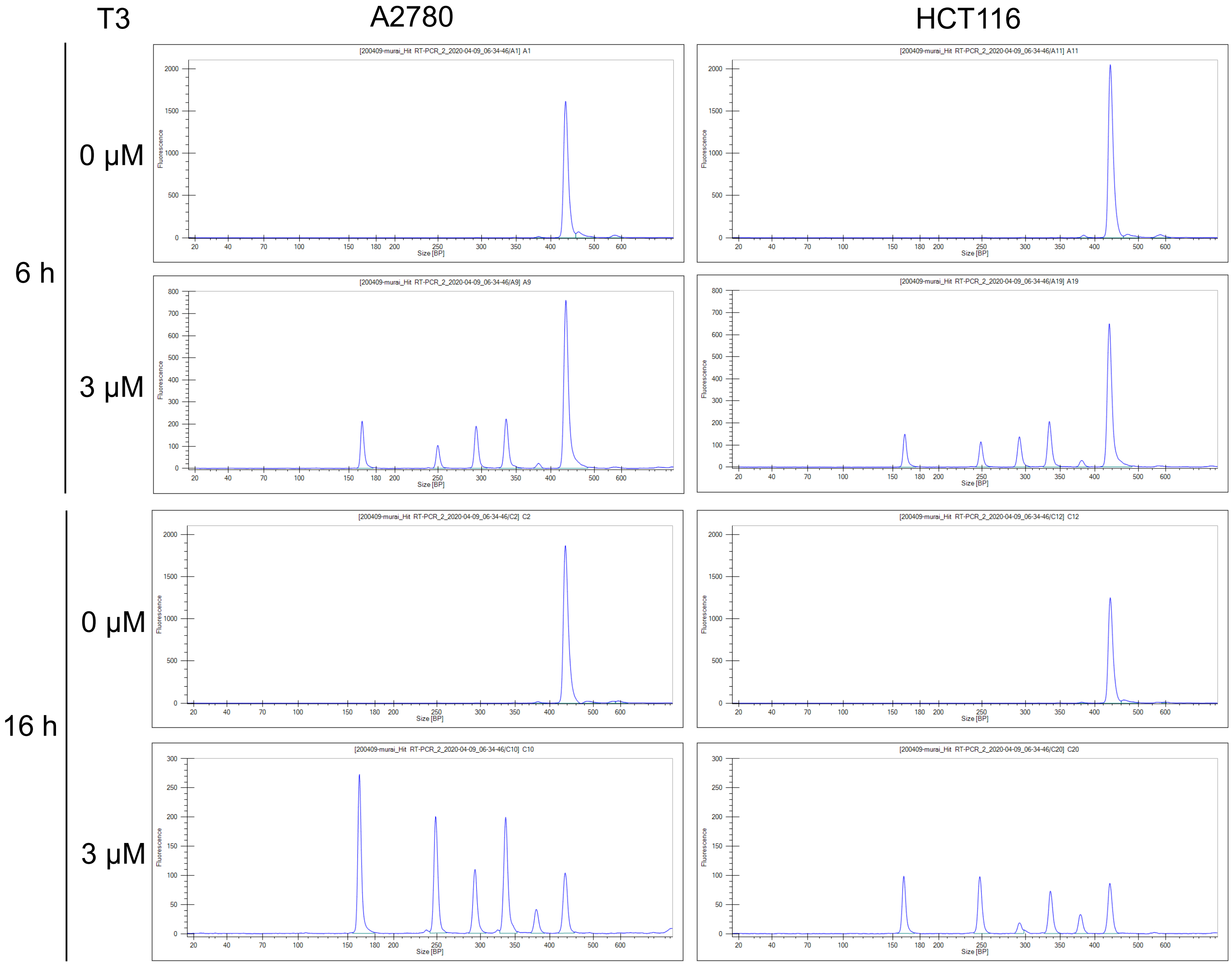

Supporting information, S4 File

Fig. 4

Raw electropherogram images using capillary electrophoresis LabChip in A2780 and HCT116 cells treated with T3.

CFLAR-S

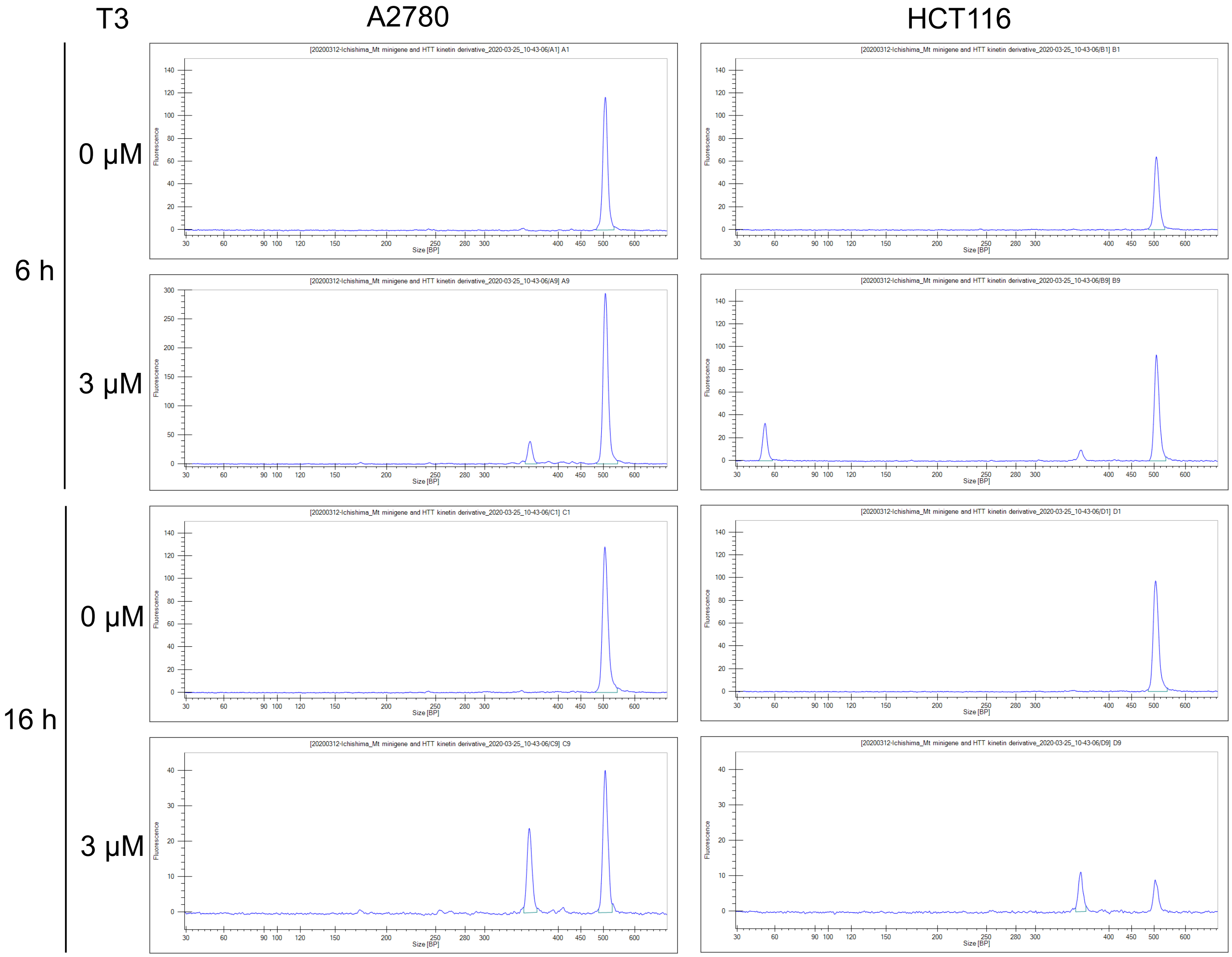

Supporting information, S4 File

Fig. 4

Raw electropherogram images using capillary electrophoresis LabChip in A2780 and HCT116 cells treated with T3.

ACTB

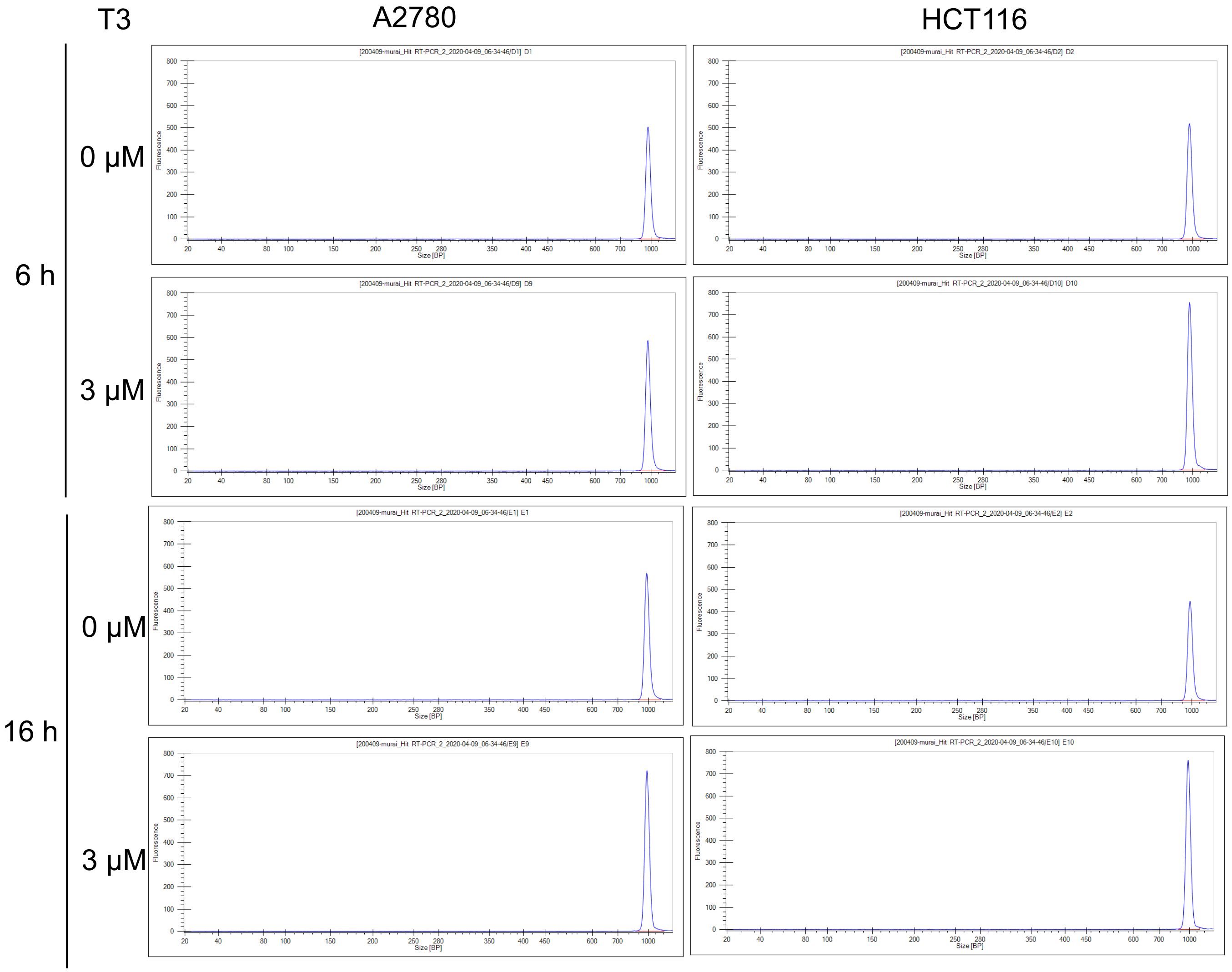

Supplement: S3 File — (PDF) [file pone.0240718.s004.pdf]
