# Supplementary material for: Synergistic apoptotic effects in cancer cells by the combination of CLK and Bcl-2 family inhibitors
Source: PLoS One. 2020 Oct 16;15(10):e0240718. doi: 10.1371/journal.pone.0240718 (PMC7567398; doi:10.1371/journal.pone.0240718)
Supplement: Attachment — Submitted filename: Response_to_reviewer_plosone_rev_f.pdf [file pone.0240718.s006.pdf]

Responses to the reviewers' comments

We thank the academic editor and the reviewers for their constructive comments and suggestions. Following the requests from the editor and reviewers, we have performed additional experiments and included descriptions of these experiments in the Introduction, Results, and Discussion sections. We believe that the manuscript has been strengthened through this process. We have responded to each of the reviewers' comments below.

Reviewer #1: The manuscript of Murai and co-authors examined the combined effects of a splicing inhibitor with an inhibitor of apoptosis for synergy in cell models of ovarian (A2780) and colorectal cancer (HCT116). In this study they present evidence that the putative CLK3 inhibitor T3 results in inhibition of splicing of anti-apoptotic proteins. Combined with an BCL-2 inhibitor (ABT-263), the authors provide evidence for synergistic increases in cell death as determined by caspase 3 assays. The authors provide further evidence that, in part, these effects are mediated through reduction of mTor signaling.

Critique
Overall these are carefully performed studies that provide initial evidence for a combinatorial approach to improve induction of cell death in cancer cells. However, there are a number of issues that need to be addressed before the manuscript is acceptable for publication.
Response:
We are grateful to Reviewer #1 for the helpful and constructive comments, and we are pleased that the reviewer considers that our studies were carefully performed. We have taken the reviewer's advice and have performed additional experiments. We respond to each of the issues raised below.

1. Only one CLK inhibitor was examined and the specificity of T3 was not discussed. The authors need to add additional experiments with another CLK inhibitor to determine if this is a drug, or target specific response. Evidence of the selectivity of T3, with respect to other kinases, needs to be provided. These studies would be more convincing if synergy was observed with other CLK inhibitors as well.
Response:

We thank the reviewer for these comments. The inhibition spectrum and phenotype selectivity of the CLK inhibitor T3 have been examined in a previous report [1]. This report found that T3 did not inhibit a panel of 71 kinases involved in multiple signaling pathways and comprehensive RNA-seq analysis of T3 treatment and knockdown of CLKs, and CLK1/2/3/4 siRNA showed 55% of RNAi alternative splicing (AS) events merged with T3-induced AS events. Studies on several CLK inhibitors have been published. However, commercially available compounds, such as TG003, are still crude, and the inhibitory activity of these compounds for CLKs is more than 50-fold weaker than T3. We do not believe that the data use compounds with concentrations above 10 µM. The available evidence strongly suggests that T3 is the most selective and potent inhibitor CLKs among the published and available compounds. As siRNAs are not suitable to use for validations with short exposure times at various concentrations, we used T3 to validate the combination study with the Bcl-2 /Bcl-xL inhibitor in this report. We have now included a description of the selectivity of T3 in the Introduction section of the article (page 4, lines 1–3).

2. The inhibition of mTOR signaling is suggested as a key part of the response although this is not well validated. Additional data demonstrating this (i.e. inhibition of S6 phos, EIF4EBP phos, or other) should be provided. At present the authors discuss the loss of S6- but no data is actually shown.

Response:

We appreciate this important comment. Koo J et al. [2] show that mTORC2 associates with SCF-FBXW7, the E3 ubiquitin ligase complex, and stabilizes MCL1 protein. mTORC2 inhibition leads to the degradation of MCL1 through the Ub-proteasome pathway. Hence, we showed the reduction of RICTOR as a result of the T3 treatment, and the results suggested its involvement in the proteasome pathway using the MG132 and T3 treatment, as shown in Fig 5A and 5B. Although inhibition of mTOR signaling is one of the essential mechanisms of action for the CLK inhibitor [3], in this report, we would like to focus on MCL1 regulation through splicing and the mTORC2-proteasome pathway. To prevent confusion, we have modified the Results section accordingly: please see page 18, line 18–page 19, line 5).

3. The explanation for the loss of MCL1 is not clear (Fig. 3C). In particular, the effects of MG132 are quite minor. Based on the PCR data shown, its possible that T3 is blocking total MCL1 expression. While the one splice (splice in) is shown, the total

MCL1 message should also be presented. The potential effects of T3 on pTEF/transcriptional regulation of MCL1 have not been ruled out.

Response:

We thank the reviewer for these comments. We have added RT-PCR data for MCL1L and MCL1S exposed to treatment with T3 in A2780 and HCT116 cells (Fig 3A). We found that T3 decreased MCL1L and induced MCL1S in a dose-dependent manner. The Percent Spliced In (PSI) value of *MCL1* was calculated as (expression of MCL1L) / (expression of MCL1L + expression of MCL1S). The PSI data revealed that T3 altered the splicing of the anti-apoptotic isoform MCL1L to the pro-apoptotic isoform MCL1S at 6 and 16 h in a dose-dependent manner (Fig. 3B). In addition to MCL1, we examined the splicing of cFLIP (*CFLAR*). The use of RT-PCR analysis to detect *CFLAR* long and short isoforms (*CFLAR*-L and -S) revealed that T3 caused a reduction in endogenously expressed *CFLAR*-L and *CFLAR*-S at 6 and 16 h, in a dose-dependent manner (Fig 4). Alternatively, T3 induced multiple smaller sized transcripts of *CFLAR*-L and *CFLAR*-S. These results strongly suggest that T3 altered the splicing of *MCL1L* and *CFLAR*, which has anti-apoptotic functions. We added this result to the Results section (page 17, lines 7–14) and Fig 3A and 4.

Reviewer #2: Authors showed that the small molecule splicing modulator T3, a CDC-like kinase (CLK) inhibitor, induced cell cycle changes and apoptosis in tumor cell lines A2780 and HCT116. T3 also showed synergistic pro-apoptotic effect with ABT-263. Authors also observed changes at protein levels for several apoptotic proteins following T3 treatment. This is an interesting study and has a potential in advancing cancer therapy. However, in the current version of this manuscript, there is a lack of direct evidence showing that the phenotypes are indeed CLK- or mRNA splicing-dependent. Limited studies on the changes in protein levels and the gene expression of only selected isoforms are not sufficient to reach the conclusion that the alternative splicing plays a critical role. Therefore, further studies are warranted before publication.

Response:

We are grateful to reviewer #2 for these insightful and positive comments, and we are pleased that the reviewer considers our work interesting and important.

1. Authors need to directly show that the phenotypes induced by T3 are CLK- or mRNA splicing-dependent. For example, cells with mutant CLK (unable to bind to T3, or splicing function deficient), or CLK knockout, or through any other means to show

that T3-induced cell cycle changes, apoptosis, and synergy with ABT-263 are CLK- or mRNA splicing-dependent. Comparing inactive T3 analog-induced vs T3–induced phenotypic changes would further help understanding the underlying mechanism.

Response:

We thank the reviewer for these comments. In a previous report, T3 shows high selectivity among 71 kinases involving multiple signaling pathways and shows phenotype selectivity using comprehensive RNA-seq analysis of T3 treatments and knockdown of CLKs [1]. CLK1/2/3/4 siRNA showed 55% of RNAi alternative splicing (AS) events merged with T3-induced AS events. Several CLK inhibitors have been published. However, commercially available compounds, such as TG003, are still crude, and the inhibitory activity of these compounds for CLKs is more than 50-fold weaker than T3. We do not believe that the data use compounds with concentrations above 10 μM. The available evidence strongly suggests that T3 is the most selective and potent inhibitor of CLKs among the published and available compounds. As the inactive T3 analog and the cells with mutant CLK were not available so far, and siRNAs are not suitable to use for validations with short exposure times at various concentrations, we used T3 to validate the combination study with the Bcl-2/Bcl-xL inhibitor in this report. We have now included a description of the selectivity of T3 in the Introduction section of the article (page 4, lines 1–3).

2. Direct evidence at mRNA levels by such as RT-PCR showing alteration of splicing events after T3 treatment should be provided for each of the splicing variants, including cFLIP-L, cFLIP-S, Mcl-1L, and Mcl-1S. Whether the mRNA splicing of those apoptotic proteins is CLK dependent also needs to be investigated.

Response:

We appreciate this comment. To address this comment, we checked the splicing of cFLIP-L, cFLIP-S, Mcl-1L, and Mcl-1S. We found that T3 altered and induced multiple splicing in the *CFLAR*-L (cFLIP-L) and *CFLAR*-S (cFLIP-S) gene loci. Endogenously expressed *CFLAR*-L and *CFLAR*-S were decreased as a result of treatment with T3, indicating that anti-apoptotic cFLIP was decreased by splicing alteration, which was consistent with the cFLIP protein level in Fig 2. Exposure to T3 decreased levels of anti-apoptotic MCL1L and induced pro-apoptotic MCL1S. These data suggest that the splicing of anti-apoptotic genes was targeted by T3. We added these results to the Results section (page 17, lines 7-14) and Fig 3A and 4.

3. Authors claimed that some of the Bcl-2 family members such as Bcl-XL and Bcl-2

were resistant to T3-induced splicing modulation. Have authors investigated other isoforms of those proteins such as Bcl-xS, Bcl2L2, etc, to support the claim?

Response:
We thank the reviewer for this comment. To address this issue, we checked the splicing of *BCL2L1* into Bcl-xS and Bcl-xL, which are major apoptotic proteins in the Bcl-2 family. T3 did not alter either Bcl-xS or Bcl-xL, indicating that the *BCl2L1* gene is resistant to T3. We have added these data to Fig 3A.

4. In Figure 3A, authors claimed "T3 altered splicing of the anti-apoptotic isoform MCL1L to the pro-apoptotic isoform MCL1S at 6 and 16 h in a dose-dependent manner", however, no data for MCL-1S has been shown. PSI for both MCL-1L and MCL-1S need to be showed.

Response:
We thank the reviewer for this comment. To address this comment, we have added RT-PCR data for MCL1L and MCL1S treated with T3 in A2780 and HCT116 cells (Fig 3A). We found that T3 decreased MCL1L and induced MCL1S in a dose-dependent manner. The Percent Spliced In (PSI) value of *MCL1* was calculated as (expression of MCL1L) / (expression of MCL1L + expression of MCL1S). The PSI data revealed that T3 altered splicing of the anti-apoptotic isoform MCL1L to the pro-apoptotic isoform MCL1S at 6 and 16 h in a dose-dependent manner (Fig. 3B). We have added this data to Fig 3A.

5. In Figure 3C, both western blot and RT-PCR for total MCl-1 and its isoforms Mcl-1L and Mcl-1S should be shown, in order to enable the comparison and delineate the effect of T3 on splicing alteration.

Response:
We thank the reviewer for this comment. To address this comment, we performed western blot and RT-PCR analyses to detect both Mcl-1L and Mcl-1S. Treatment with T3 led to a modest decrease in Mcl-1S, but the extent of the decrease was not remarkable when compared to Mcl-1L (Fig 2). Mcl-1L was completely deleted after treatment with 3 µM of T3 in HCT116 cells, whereas Mcl-1S was expressed in the same condition. RT-PCR analysis revealed that T3 decreased Mcl-1L and induced Mcl-1S in a dose-dependent manner (Fig 3A). These results suggest that there is a discrepancy between the protein and transcript levels of the MCL1 isoform. As shown in Fig 5, it has been suggested that T3 also reduces the Mcl-1 (Mcl-1L and Mcl-1S) protein via the mTORC2-regulated proteasome pathway. Presumably, the reduction of the Mcl-1

protein by T3 is regulated by both proteasome and splicing alterations. We have added these data (Fig 2 and 3A) and some text regarding these analyses to the Results (page 15, line 14–page 16, line 4) and Discussion (page 23, lines 1–4) sections of the article.

6. A section of statistical analysis should be included.

Response:

We are grateful for this suggestion. We have improved the manuscript by including a section on statistical analysis in the Materials and methods section: please see page 11, line 15 to page 12, line 2.

References

1. Funnell T, Tasaki S, Oloumi A, Araki S, Kong E, Yap D, et al. CLK-dependent exon recognition and conjoined gene formation revealed with a novel small molecule inhibitor. Nat Commun. 2017;8(1):7. Epub 2017/02/25. doi: 10.1038/s41467-016-0008-7
10.1038/s41467-016-0008-7 [pii]. PubMed PMID: 28232751.
2. Koo J, Yue P, Deng X, Khuri FR, Sun SY. mTOR Complex 2 Stabilizes Mcl-1 Protein by Suppressing Its Glycogen Synthase Kinase 3-Dependent and SCF-FBXW7-Mediated Degradation. Mol Cell Biol. 2015;35(13):2344-55. Epub 2015/04/29. doi: MCB.01525-14 [pii]
10.1128/MCB.01525-14. PubMed PMID: 25918246; PubMed Central PMCID: PMC4456440.
3. Araki S, Dairiki R, Nakayama Y, Murai A, Miyashita R, Iwatani M, et al. Inhibitors of CLK protein kinases suppress cell growth and induce apoptosis by modulating pre-mRNA splicing. PLoS One. 2015;10(1):e0116929. Epub 2015/01/13. doi: 10.1371/journal.pone.0116929
PONE-D-14-35737 [pii]. PubMed PMID: 25581376; PubMed Central PMCID: PMC4291223.
