# Supplementary material for: Synergistic apoptotic effects in cancer cells by the combination of CLK and Bcl-2 family inhibitors
Source: PLoS One. 2020 Oct 16;15(10):e0240718. doi: 10.1371/journal.pone.0240718 (PMC7567398; doi:10.1371/journal.pone.0240718)
Supplement: Attachment — Submitted filename: Responses_to_reviewers_rev2_f.pdf [file pone.0240718.s007.pdf]

Responses to reviewers’ comments

We thank the academic editor and reviewers for their constructive comments and suggestions. According to the suggestions provided by the editor and reviewers, we performed additional experiments and included descriptions of these experiments in the Introduction, Results, and Discussion sections. The reviewers’ comments and our responses are listed below.

Reviewer #1: Please revise sentence on p.4 line 1-does not currently make sense. The digitized blots in Figs 3 and 4 are unacceptable.

Response:

We have revised the description of page 4, line 1.

Digitized blots in Fig. 3 were replaced with quantified data of MCL1 and BCL2L1 normalized to *ACTB*. We have added the raw electropherogram data related to Fig. 4 into Supplemental Information section (S4 File) because showing both data in one figure would have resulted in a huge file.

Reviewer #2: Authors did not directly and experimentally address many major concerns the reviewer has raised. One major point is the specificity aspect. Even though T3 has been reported to be potent, the off-target binding is still a possibility unless proven otherwise. Both the reviewers have specifically indicated that additional experiments with pharmacological and/or genetic approaches should be performed in order to show that the effect is CLK- or splicing-dependent. Unfortunately, authors did not attempt to address this point experimentally.

Response:

We performed additional experiments and added several descriptions to address the concerns raised. The possibility of off-target binding was assessed using an inactive analog, T3-1, with a fluoro group at the 8-position T3 (Fig S1). T3-1 did not show inhibitory activity for CLK1 or CLK2. Considering the previous docking model study of T3 [1], T3-1 might not bind to the hinge region of CLK2. T3-1 did not inhibit cell growth even at 10 µM and did not show any apoptotic synergistic effect with Bcl-2/Bcl-xL inhibitor (Fig S1 and Fig 6). These data support a CLK-dependent phenotype for T3. We have added these data (Fig 6 and Fig S1) and some text regarding these analyses to the Results section (page 13, lines 7–11 and page 21, lines 3–4).

Figure 3A showed that the lower molecular weight BCL2L1 isoform Bcl-xS increased dose-dependently upon T3 treatment up to 1 uM, which is especially apparent after 16h treatment in both cell lines, and the amount of change is similar to MCL1 but with a different kinetics. However, authors described T3 "had no effect on splicing of the BCL2L1 gene". This causes confusion.

Response:

We revised Fig. 3A to show *BCL2L1* and *MCL1*, normalized to *ACTB* to avoid confusion. The expression of *MCL1L* and *MCL1S* increased and decreased, respectively, in a dose-dependent manner after treatment with T3. The short isoform of *BCL2L1* (*BCL2L1S*) coding Bcl-xS was induced by 1-3 µM of T3; however, 10 µM of T3 did not induce *BCL2L1S*. T3 also did not alter splicing of the long form of *BCL2L1* (*BCL2L1L*) coding Bcl-xL. These data reflect percent spliced in (PSI) index data, normalized by relative expression of short and long forms in *MCL1* or *BCL2L1*. Data indicate that T3 alters splicing of *MCL1*, but not of *BCL2L1* (Fig. 3B). We have revised the description in the Results section (page 17, lines 1–6) to avoid confusion.

There is also a considerable confusion regarding cFLIP. Authors stated that "T3 induced smaller size of cFLIPL and larger size of cFLIPL", and then "T3 induced the smaller sized cFLIPL and cFLIPS", and also stated "T3 alternatively induced multiple smaller sized transcripts of CFLAR-L and CFLAR-S". In fact, the Fig 2 showed that T3 induced LARGER size of cFLIPs protein, but in Fig 4 there is NO LARGER size of cFLAR-S RNA but only SMALLER size cFLAR-S RNA. Would authors be able to consolidate?

Response:

We apologize for the confusion. Immuno-blot analysis revealed that T3 induced different sizes of $cFLIP_L$ and $cFLIP_S$. We have not yet clarified the origin– $cFLIP_L$ or $cFLIP_S$–of the different sized bands. RT-PCR analysis showed that T3 alternatively induced multiple smaller sized transcripts of *CFLAR-L* and *CFLAR-S*, suggesting that T3 altered the splicing of *CFLAR*. We revised these descriptions in the Results section (page 16, lines 5–7 and page 17, lines 12–17).

Upon T3 treatment, Mcl-1S increased at RNA level (Fig 3A) but decreased at protein level (Fig 2). Reviewer's Point 5 suggested to study the MCl-1 isoforms in Fig 3C (the original version), with using MG132 to investigate if the discrepancy in RNA and protein levels is at least due to proteasome degradation. Would the levels of Mcl-1S RNA become consistent with its protein levels if MG132 is present? Unfortunately, authors did not address this point experimentally.

Response:

We performed additional immuno-blot analysis to detect both Mcl-1L and Mcl-1S using co-treatment with MG-132 with or without T3. MG-132 treatment without T3 induced Mcl-1L; however, MG-132 did not induce Mcl-1S (Fig 5B). Co-treatment with MG-132 and T3 decreased both Mcl-1L and Mcl-1S proteins. MG-132 induced Mcl-1L and slightly induced Mcl-1S proteins in the presence of 3 μM T3. These results imply that Mcl-1S is slightly degraded by the proteasome pathway upon treatment with T3. These data may partially explain the discrepancy between RNA and protein levels of Mcl1-S after T3 treatment. We have added these data (Fig 5B) and some text regarding these analyses to the Results section (page 19, lines 12–16)

Reference

1. Funnell T, Tasaki S, Oloumi A, Araki S, Kong E, Yap D, et al. CLK-dependent exon recognition and conjoined gene formation revealed with a novel small molecule inhibitor. Nat Commun. 2017;8(1):7. Epub 2017/02/25. doi: 10.1038/s41467-016-0008-7
10.1038/s41467-016-0008-7 [pii]. PubMed PMID: 28232751; PubMed Central PMCID: PMC5431906.
