# Supplementary material for: Synergistic apoptotic effects in cancer cells by the combination of CLK and Bcl-2 family inhibitors
Source: PLoS One. 2020 Oct 16;15(10):e0240718. doi: 10.1371/journal.pone.0240718 (PMC7567398; doi:10.1371/journal.pone.0240718)
Supplement: Attachment — Submitted filename: The manuscript of Murai and co.docx [file pone.0240718.s005.docx]

The manuscript of Murai and co-authors examined the combined effects of a splicing inhibitor with an inhibitor of apoptosis for synergy in cell models of ovarian (A2780) and colorectal cancer (HCT116). In this study they present evidence that the putative CLK3 inhibitor T3 results in inhibition of splicing of anti-apoptotic proteins. Combined with an BCL-2 inhibitor (ABT-263), the authors provide evidence for synergistic increases in cell death as determined by caspase 3 assays. The authors provide further evidence that, in part, these effects are mediated through reduction of mTor signaling.

Critique

Overall these are carefully performed studies that provide initial evidence for a combinatorial approach to improve induction of cell death in cancer cells. However, there are a number of issues that need to be addressed before the manuscript is acceptable for publication.

1. Only one CLK inhibitor was examined and the specificity of T3 was not discussed. The authors need to add additional experiments with another CLK inhibitor to determine if this is a drug, or target specific response. Evidence of the selectivity of T3, with respect to other kinases, needs to be provided. These studies would be more convincing if synergy was observed with other CLK inhibitors as well.

2. The inhibition of mTOR signaling is suggested as a key part of the response although this is not well validated. Additional data demonstrating this (i.e. inhibition of S6 phos, EIF4EBP phos, or other) should be provided. At present the authors discuss the loss of S6- but no data is actually shown.

3. The explanation for the loss of MCL1 is not clear (Fig. 3C). In particular, the effects of MG132 are quite minor. Based on the PCR data shown, its possible that T3 is blocking total MCL1 expression. While the one splice (splice in) is shown, the total MCL1 message should also be presented. The potential effects of T3 on pTEF/transcriptional regulation of MCL1 have not been ruled out.
